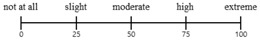# Effects of a Motion Seat System on Driver’s Passive Task-Related Fatigue: An On-Road Driving Study

**DOI:** 10.3390/s20092688

**Published:** 2020-05-08

**Authors:** Seunghoon Lee, Minjae Kim, Hayoung Jung, Dohoon Kwon, Sunwoo Choi, Heecheon You

**Affiliations:** 1 Department of Industrial and Management Engineering, Pohang University of Science and Technology, Pohang 37673, Korea; shooonlee@postech.ac.kr (S.L.); minjae@postech.ac.kr (M.K.); niceterran36@postech.ac.kr (H.J.); kydson@postech.ac.kr (D.K.); 2 Body Test Team 3, Hyundai Motor Company, Hwaseong 18280, Korea; csw@hyundai.com

**Keywords:** passive task-related driver fatigue, monotonous driving, fatigue countermeasure, motion seat system

## Abstract

Passive task-related (TR) fatigue caused by monotonous driving can negatively affect driving safety by impairing driver alertness and performance. This study aims to evaluate the effectiveness of a motion seat system on the driver’s passive TR fatigue in terms of driving performance, physiological response, and subjective fatigue by using automotive and physiological sensors those applicable to on-road driving environment. Twenty drivers (5 females and 15 males; age = 38.5 ± 12.2) with more than two years of driving experience participated in an on-road experiment with two driving conditions: driving in the static seat condition during the first half of the driving session and then in the static (static–static, SS) or motion seat (static–motion, SM) condition during the second half. The SM condition showed significantly lower passive TR fatigue by 4.4~56.5% compared to the SS condition in terms of the standard deviation of velocity, percentage of eyelid closure rate (PERCLOS), and the ratio of low- to high-frequency power (LF/HF) of electrocardiography signals. The drivers rated significantly lower subjective state changes of overall fatigue, mental fatigue, passive TR fatigue, drowsiness, and decreased concentration in the SM condition than those in the SS condition. The findings of the study support the use of a motion seat system can be an effective countermeasure to reduce passive TR fatigue.

## 1. Introduction

Driver fatigue can cause traffic accidents by degrading the driver’s alertness and performance. Driver fatigue is an impaired state of mental alertness, negatively affecting cognitive and psychomotor functions such as visual–spatial ability, memory, information processing, and rapid reaction required for driving tasks [1,2,3]. Driver fatigue can be classified into active task-related (TR) fatigue in cognitive overload and passive TR fatigue in cognitive underload [4,5]. For example, active TR fatigue can occur in a high workload situation such as driving in high-density traffic or poor visibility environment, while passive TR fatigue in a low workload situation such as driving on a monotonous highway or in a partially autonomous vehicle [1,4]. Existing studies have reported that 20~35% of road accidents are due to driver fatigue [6,7,8]; for example, Hartley [8] reported that driver fatigue is attributable to 35% of all fatal crashes in rural areas and 12% of those in urban areas.

Passive TR fatigue has been examined in terms of driving performance and the driver’s workload using automotive and physiological sensors. Automotive sensors of vehicle speed [9,10,11], lane position [9,10,12], and steering wheel angle [9,13,14] have been widely used to examine the effects of the driver’s passive TR fatigue on the driver’s ability to control the vehicle. Next, physiological sensors such as electrocardiogram (ECG) [9,15], eye tracker [16,17], skin conductance (SC) [18,19,20], and electroencephalogram (EEG) [9,16,18,21,22] have been used to examine the effects of the driver’s passive TR fatigue on the driver’s physiological state. For example, passive TR fatigue can result in increased heart rate variability (HRV) [23] and eye blink duration [21,24], decreased skin conductance level [18,20], and increased EEG powers in the *α* (0.5~4 Hz) and *θ* (8~13 Hz) bands [22,25]. Proper automotive and physiological sensors can be selected for an on-road driving study by considering driving safety, robustness from noise, and comfort. For example, steering wheel reversal rate can be excluded due to high possibility of a traffic accident, EEG due to high sensitivity to noise in an on-road driving condition [18], and SC due to interference of SC electrodes attached to the fingers with the operation of the steering wheel.

The effectiveness of various interactive technologies to mitigate passive TR fatigue by increasing mental alertness during monotonous driving has been examined mostly in the lab experiments due to safety concerns. Verwey and Zaidel [26] demonstrated that a speech-controlled game box system which provides a secondary task such as Tetris during driving decreased subjective drowsiness by 14.6% and the number of sleep-related errors including accidents and line crossing by 44.4% compared to the normal driving condition. Gershon et al. [27], Oron-Gilad et al. [9], and Song et al. [10] reported that an interactive cognitive task such as trivia games could be an effective countermeasure to prevent the performance decrease of a driver during monotonous driving. Schmidt et al. [28] reported that 15 °C thermal stimuli directed to the face of a driver from the air vents of the center fascia increased the pupil diameter and significantly decreased the sleepiness level by 0.5~0.6 points using the 9-point Karolinska Sleepiness Scale [29]. Researchers including the authors of this study identified that a motion seat system which changes the seat configuration periodically helped drivers maintain their performance in brake reaction time, the standard deviation (SD) of lane position, and percentage of eyelid closure rate (PERCLOS) during a 90-min monotonous driving in the lab environment, while a conventional seat system did not (increases of 92.8 msec in brake reaction time, 6.0 cm in SD of velocity, and 1.3% of PERCLOS in the second 45-min driving session from those in the first 45-min driving session) [30].

On-road driving evaluation is needed to ensure the validity of the passive TR fatigue reduction effect of interactive technology. A driving scenario with low variability in roadside scenery and traffic flow is administered in a lab experiment to induce passive TR fatigue from the driver [9,11,13,18,31]. However, it is challenging to cause passive TR fatigue in an on-road experiment because on-road driving often requires a high level of vigilance from the driver or various environmental factors such as a sudden change in traffic or weather can affect the level of vigilance by acting as external stimuli [9,32,33]. For example, Philip et al. [34] reported that inappropriate line crossing was increased by prolonged driving in the simulation driving condition, but no significant changes in driving performance could be observed in the first 2 h of on-road driving because the driver might allocate more attention to possible hazardous events. Lastly, de Winter et al. [35] discussed that simulated driving environments might induce demotivation or unrealistic driving behaviors from participants due to lacking fidelity.

The present study is to examine the effects of a motion seat system on passive TR fatigue in terms of driving performance, physiological response, fatigue behavior, and subjective fatigue in an on-road driving environment. A 90-min monotonous driving task was administered on a highway with low traffic to induce passive TR fatigue from drivers. The longitudinal velocity and steering wheel rate of the vehicle and the HRV, PERCLOS, facial expressions, and gestures of the driver were recorded during the driving; subjective fatigue was evaluated before and after the driving using a multidimensional driver fatigue evaluation questionnaire.

## 2. Materials and Methods

### 2.1. Participants

Twenty Korean participants (male: 15, female: 5) in their 20s to 50s (mean ± *SD* = 38.5 ± 12.2; *range* = 24~63 years) with more than two years of driving experience (mean ± *SD* = 14.0 ± 12.2 years; *range* = 3~35 years) participated in the on-road driving evaluation. Healthy participants without cardiovascular and musculoskeletal disorders were recruited to minimize changes caused by factors other than fatigue. The participants were asked to keep from drinking alcohol and caffeine for 24 h before the experiment [15,17] and sleep more than 8 h on the day before the driving experiment. The present study was approved by the Institutional Review Board (IRB) of the Pohang University of Science and Technology (PIRB-2018-E080).

### 2.2. Apparatus

**Figure 1:**
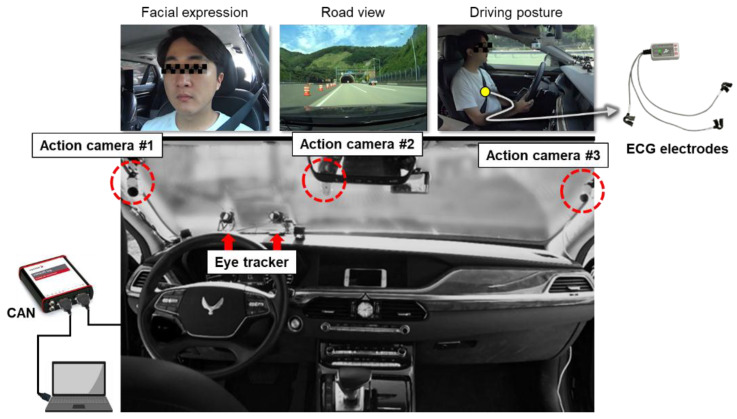
Driver cockpit for the on-road evaluation of passive task-related fatigue of the driver.

**Figure 2:**
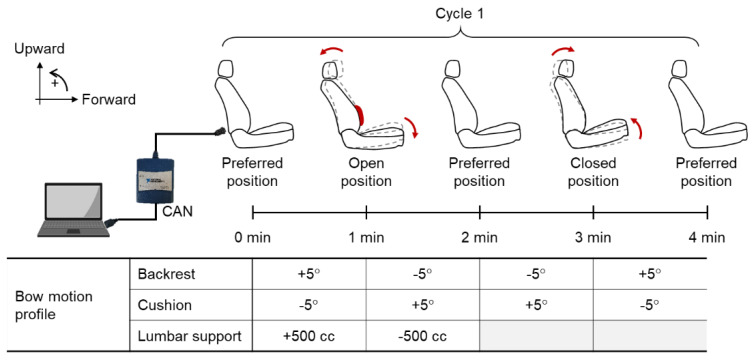
Designated seat motion profile of backrest recline, cushion tilt, and lumbar support inflation/deflation.

As shown in Figure 1, a vehicle of G90 (Hyundai-Kia Motors, Republic of Korea) with a motion seat system was retrofitted to the on-road driving evaluation in the present study. The motion seat system was configured by connecting the electronic control unit (ECU) of the driver seat and a PC using the USB-8473 controller area network (CAN) interface device (National Instruments, USA) to control the configuration of the seat. The motion seat system was able to control the coordinated motions of the backrest recline, cushion tilt, and lumbar support inflation/deflation at a one-minute time interval following a designated motion profile to induce the stretching and flexion of the whole body. As shown in Figure 2, the motion profile was designed for the seat to move cyclically from the preferred seat position of the driver to open and closed positions. The open position, which induces the extension of the body, was configured by a recline (say, 5°) of the backrest, a downward tilt (say, 5°) of the cushion, and an inflation level (say, 500 cc) of the lumbar support to stretch the body. The closed position, which induces the flexion of the body, was configured by the opposite motion. Measurements of driving performance such as longitudinal velocity and steering wheel rate were transmitted from the ECU of the vehicle to the PC at 250 Hz using the VN1630A CAN interface device (Vector, Germany) and Vehicle Network Toolbox^TM^ (MathWorks Inc., Natick: MA, USA). Finally, the driving assistance systems of the vehicle, such as adaptive cruise control and lane-keeping assistance systems, were deactivated to observe the decrease in the driving performance of the driver over the driving session.

The wireless ECG measurement system DTS BioMonitor XPTM (NORAXON Inc., USA, sampling rate; 1500 Hz) and the eye tracker faceLAB 5 (Seeing Machines Inc., USA; sampling rate = 60 Hz) were used to obtain HRV and PERCLOS data of the driver during the driving experiment (see Figure 1). HRV was quantified by low- to high-frequency power (LF/HF) in the frequency domain using RR interval measurements of ECG signals extracted using the QRS detection algorithm of Pan and Tompkins [36]. Artifacts of RR interval measurements such as ectopic beats and arrhythmic events were removed by detecting the abnormal R-peak which appeared within the next 350 ms after the adjacent R-peak [36]. RR interval measurements were interpolated via cubic spline and re-sampled at 5.69 Hz (1024 samples per 3-min time window) to create a uniformly spaced time series for spectral HRV analysis [37] because the RR interval measurements were not evenly sampled and hence fast Fourier transform (FFT) algorithm could not be used directly. LF/HF was calculated as the ratio of low-frequency band (0.04–0.15 Hz) power to high-frequency band (0.15–0.4 Hz) power by applying the FFT algorithm to RR interval measurements by Equation (1). The eye tracker mounted on the dashboard without interfering with the driver’s visibility was calibrated for eye activity measurement with an error of less than 2°. PERCLOS was calculated as the percentage of time in which the driver’s eyelids are closed for more than 75% over a 3-min moving window [16,17,38].

(1) $$\mathrm{LF}/\mathrm{HF}=\int_{0.04Hz}^{0.15Hz}f\left(\lambda \right)d\lambda /\int_{0.15Hz}^{0.40Hz}f\left(\lambda \right)d\lambda ,$$

where: f(λ)dλ = power spectrum of the RR tachogram.

Three action cameras of HDR-AS200V (SONY Inc., Japan; sampling rate = 60 Hz) were used to record the fatigue behaviors of the driver and driving environments. The action cameras were installed to the A-pillars of the driver and passenger sides and the windshield next to the room mirror to observe the driver’s facial expressions, gestures, and driving environments (see Figure 1). The two action cameras at the A-pillars recorded facial expressions such as eye blinking, yawning, and head motions such as head shaking and face scratching; the action camera at the windshield recorded the driving environments in front of the vehicle to exclude measurements during situations interrupting monotonous driving (e.g., construction areas, traffic congestion areas, and lane change areas) from the analysis. The fatigue status of the driver was assessed by three ergonomists into one of three fatigue status categories (awake, drowsy, and very drowsy) by observing video clips segmented at a three-minute interval according to the driver fatigue level criteria (see Table 1) [39]. Lastly, CAN signals, ECG signals, eye-tracking data, and action camera video-recordings with different sampling rates were synchronized.

A multidimensional driver fatigue evaluation questionnaire (Table 2) developed in the present study was used to evaluate the driver’s passive TR fatigue. Multidimensional questionnaires such as Swedish Occupational Fatigue Inventory-20 (SOFI; Ashberg [40]) and Dundee Stress State Questionnaire (DSSQ; Matthews et al. [41]) were used to measure various fatigue behaviors caused by monotonous driving [9,24,42]. In this study, subjective fatigue was further divided into overall fatigue, physical fatigue (e.g., muscle pain and eye strain), and mental fatigue (e.g., boredom and decreased alertness); mental fatigue into active TR fatigue caused by active driving tasks (e.g., pedal and steering wheel operations) and passive TR fatigue caused by sustained attention tasks. Lastly, passive TR fatigue was further divided into decreases in motivation, energy arousal, and concentration of task engagement, which were selected from DSSQ as those related to passive TR fatigue [43,44,45]. A visual analog scale (VAS) consisting of five anchors (0 = not at all, 25 = slight, 50 = moderate, 75 = high, and 100 = extreme) was employed to the driver fatigue evaluation questionnaire. A driver fatigue change was calculated to a state change (z) by subtracting a VAS rating before driving from a VAS rating after driving. Since the levels of subjective fatigue can be assessed differently within the participant or between the participants even if the fatigue levels were identical, a state change was normalized by the standard deviation of the state scores obtained before driving, which was employed by Matthews et al. [43] for the DSSQ analysis.

### 2.3. Experimental Procedure

**Figure 3:**
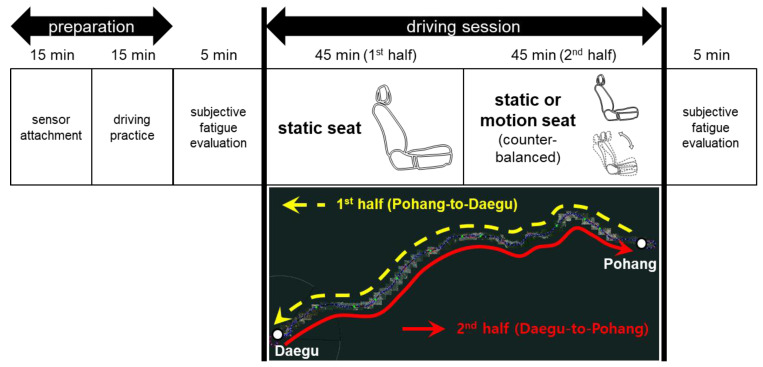
Experimental procedure of an on-road evaluation of the driver’s passive task-related fatigue.

The experiment of on-road motion seat system evaluation was conducted in four phases: (1) preparation, (2) subjective fatigue evaluation before driving, (3) main experiment, and (4) subjective fatigue evaluation after driving (Figure 3). First, in the preparation phase, informed consent was obtained after explaining the purpose and procedure of the experiment to the participant. The participant was allowed to adjust to his/her preference the fore-aft and height of the seat, recline angle of the seatback, and tilt of the cushion for comfortable posture and the side and review mirrors for proper visibility. The eye cameras were calibrated based on the eye position of the participant; ECG electrodes were attached to the left clavicle, right clavicle, and stomach of the participant. A 15-min driving practice session was provided for the participant to become familiarized with the driving task. Second, subjective fatigue before driving was evaluated using the driver fatigue evaluation questionnaire. Third, in the main experiment phase, a monotonous driving task of maintaining 90 to 100 km/h while staying in the second lane on the highway was conducted in the static and motion seat conditions. The 134-km-long highway of Daegu to Pohang mostly consists of straight two-lane roads with occasional moderate curves. Note that a preliminary experiment in the present study confirmed that the driving environment of the Daegu-Pohang highway was proper for monotonous driving because of its low traffic density and similar scenery. The driving experiment was administered from noon to six o’clock in the afternoon when the weather is fair without rain to ensure proper driving conditions and safety. The driving task was performed in the static seat condition during the first half of the driving session to induce passive TR fatigue from the participant and then was continued in the static (static–static, SS) or motion seat (static–motion, SM) condition during the second half of the driving session (Figure 3). Vehicle speed, steering wheel rate, ECG parameters, and eyelid closure rate of the participant were measured while driving. A researcher accompanied the participant in the front passenger seat to observe the fatigue status of the participant and driving environment changes; the experiment was discontinued if the driver’s fatigue was significantly high so that driving safety could be compromised. Lastly, subjective fatigue after driving was evaluated. The SS and SM conditions (one day for each condition) were counterbalanced.

### 2.4. Statistical Analysis

The measures of driving performance, physiological responses, and driving behaviors between the first-half and second-half sessions of the 90-min driving were compared for the SS and SM conditions. Passive TR fatigue data of 19 participants except for one driver who showed the severe drowsiness level during the driving task was analyzed. The detour section (61 km to 70 km from Pohang) with high variability of roadside scenery and traffic flow was excluded due to relatively low monotony compared to the first- and second half of the driving session. Repeated measures ANOVA were performed at the significance level *α* = 0.05 to assess the effectiveness of one within-subject factor of the seat condition. The normality assumption of the test variables were confirmed by Shapiro-Wilk test [46]. SPSS v. 18.0 (International Business Machines INC., USA) was used for the statistical analysis.

## 3. Results

### 3.1. Driving Performance

**Figure 4:**
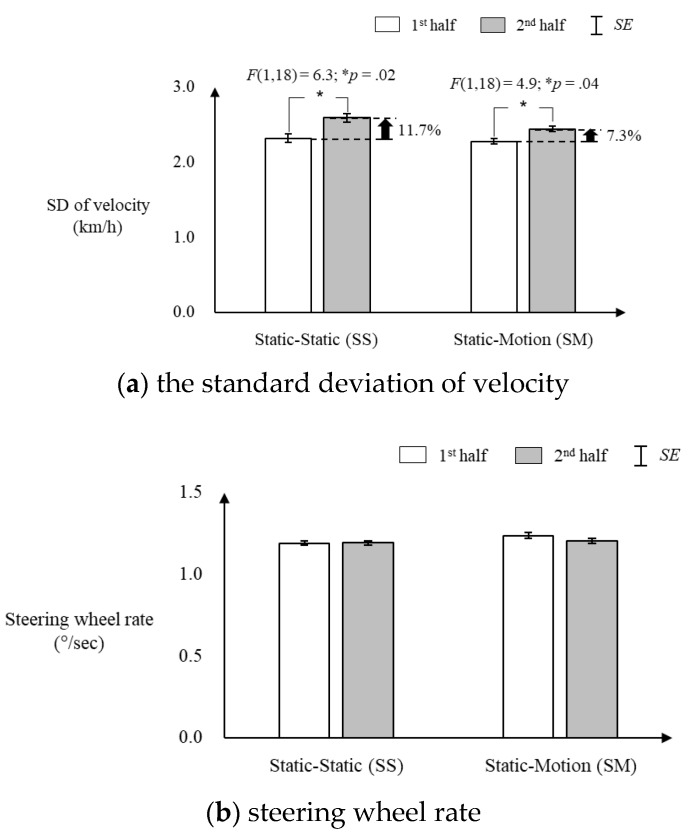
Changes in driving performance between the first-half and second-half driving sessions for the static (S) and motion (M) seat conditions.

The SD of velocity significantly increased over the driving sessions for both the SS and SM conditions, while no significant difference was observed in steering wheel rate regardless of seat condition. Figure 4a shows that the SD of velocity in the SS condition significantly increased by 11.7% in the second-half (*M* ± *SE* = 2.59 ± 0.05 km/h) driving session compared to that in the first-half (*M* ± *SE* = 2.32 ± 0.05 km/h) (F [1,18] = 6.3, *p* = 0.02), while the SM condition increased by 7.3% (*M* ± *SE* = 2.28 ± 0.04 km/h in first-half and 2.44 ± 0.04 km/h in second-half) (F [1,18] = 4.9, *p* = 0.04). Figure 4b shows that no significant difference in steering wheel rate was found between the first-half and second-half sessions for all the SS and SM conditions (*p* > 0.05).

### 3.2. Physiological Indicators

**Figure 5:**
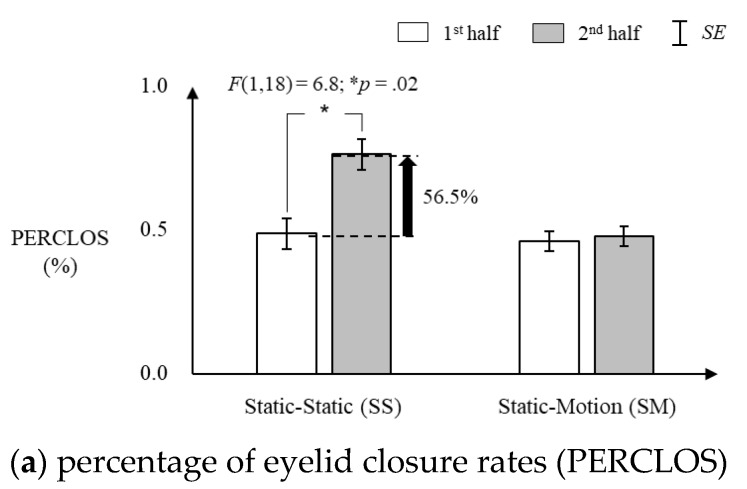

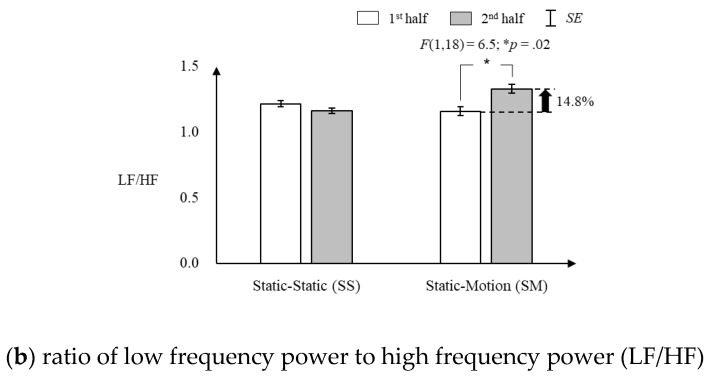
Changes in physiological responses between the first-half and second-half driving sessions for the static (S) and motion (M) seat conditions.

PERCLOS significantly increased in the SS condition but was maintained in the SM condition, while LF/HF was maintained in the SS condition but significantly increased in the SM condition over the driving sessions (Figure 5). Figure 5a shows that PERCLOS in the SS condition significantly increased by 56.5% in the second-half (*M* ± *SE* = 0.76% ± 0.05%) driving session compared to that in the first-half (*M* ± *SE* = 0.49% ± 0.05%) (F [1,18] = 6.8, *p* = 0.02). Next, Figure 5b shows that LF/HF in the SM condition significantly increased by 14.8% in the second-half (*M* ± *SE* = 1.33 ± 0.03) driving session compared to that in the first half (*M* ± *SE* = 1.16 ± 0.03) (F [1,18] = 6.5, *p* = 0.02).

### 3.3. Number of Fatigue Behaviors

**Figure 6:**
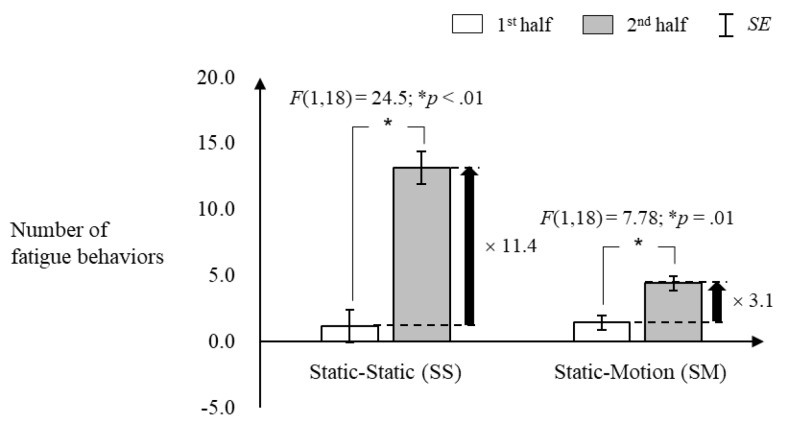
Number of fatigue behaviors.

The number of fatigue behaviors increased less in the SM condition than in the SS condition over the driving sessions. All the observed fatigue statuses (see Table 1) did not exceed level 2 (drowsy) because the experiment was planned to terminate by the researcher before the fatigue level of the participant was aggravated more than level 2. As shown in Figure 6, the number of fatigue behaviors in the first-half driving session was similar among the SS (1.16 ± 1.21 times) and SM conditions (1.42 ± 0.54 times) (*p* > 0.05), but increased 11.4 times for the SS condition (F [1,18] = 24.5, *p* < 0.01) and 3.1 times for the SM condition (F [1,18] = 7.8, *p* = 0.01) in the second-half driving session compared to those in the first-half.

### 3.4. Subjective Fatigue

**Figure 7:**
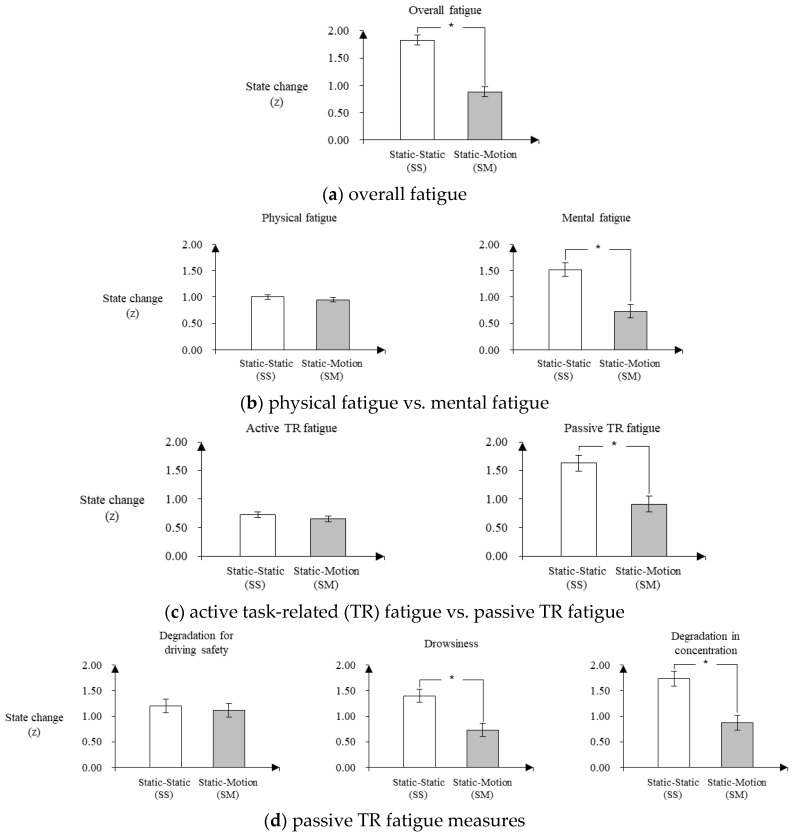
Fatigue state changes between the before- and after-driving sessions for the static- and motion-seat conditions.

As shown in Figure 7, the state changes of all the subjective fatigue measures except physical fatigue, active TR fatigue, and degradation of driving safety were significantly lower in the SM condition than those in the SS condition. Figure 7a shows that the state change of overall fatigue in the SM condition (*M* ± *SE* = 0.88 ± 0.09) was significantly lower by 0.95 than that in the SS condition (*M* ± *SE* = 1.83 ± 0.09) (F [1,18] = 31.4, *p* < 0.01). Figure 7b exhibits that the state change of mental fatigue in the SM condition (*M* ± *SE* = 0.73 ± 0.13) was significantly lower by 0.79 than that of the SS condition (*M* ± *SE* = 1.52 ± 0.13) (F [1,18] = 11.0, *p* < 0.01), while no significant difference in the state change of physical fatigue between the SS and SM conditions was found (F [1,18] = 0.5, *p* = 0.50). Next, Figure 7c displays that the state change of passive TR fatigue in the SM condition (*M* ± *SE* = 0.91 ± 0.14) was significantly lower by 0.72 (F [1,18] = 7.4, *p* = 0.01), while no significant difference in the state change of active TR fatigue was observed between the SS and SM conditions (*F* [1,18] = 0.6, *p* = 0.46). Lastly, Figure 7d shows that the state changes of drowsiness (*M* ± *SE* = 1.40 ± 0.13 in SS condition and 0.73 ± 0.13 in SM condition) (F [1,18] = 7.8, *p* = 0.01) and degradation in concentration (*M* ± *SE* = 1.73 ± 0.15 in SS condition and 0.87 ± 0.15 in SM condition) (F [1,18] = 9.8, *p* < 0.01) in the SM condition were significantly lower compared to those in the SS condition, while no significant difference in the state change of degradation of driving safety was found between the SS and SM conditions (F [1,18] = 0.1, *p* = 0.72).

## 4. Discussion

The on-road driving evaluation of the present study found the motion seat system effective in reducing passive TR fatigue caused by monotonous driving. First, from the aspect of driving performance, the increased percentage of the SD of velocity over the first-half and second-half driving sessions was 4.4% lower in the SM condition compared to that of the SS condition, which indicates that the motion seat system was effective in reducing the deterioration of driving performance. Ting et al. [11] reported that the SD of velocity progressively increased by time-on-task during the 90-min monotonous driving task and had a high correlation (*r* = 0.858) with reaction time, which is closely related to driving safety [21,24,34,42]. Second, from the aspect of physiological responses, PERCLOS was kept leveled and LF/HF significantly increased in the second-half driving session compared to those in the first-half for the SM condition, while the opposite was observed for the SS condition, which indicates that the motion seat system was effective in reducing drowsiness and increasing alertness. Schömig et al. [47] and Jarosch et al. [48] reported that solving quiz questions on a tablet increased the cognitive workload of the driver in an automated driving condition by resulting in a significant decrease in PERCLOS (the higher the PERCLOS, the higher the drowsiness). Next, Zhang et al. [49] reported that 4~7 Hz of vibration transmitted to the whole body from the seat while driving on a monotonous highway enhanced the alertness of the driver by increasing LF/HF [15,50]. Third, from the aspect of fatigue behavior, the number of fatigued driving behaviors increased much less in the SM condition than in the SS condition over the first-half and second-half driving sessions. Lastly, from the aspect of subjective fatigue, the state changes of all the subjective fatigue measures except physical fatigue, active TR fatigue, and degradation of driving safety were significantly lower in the SM condition than those in the SS condition. The increases in the subjective fatigue measures observed in the present study were consistent with the findings of the existing studies, indicating that subjective fatigue was significantly increased also in low-workload situations such as driving in a monotonous environment [9,26] or a partially autonomous vehicle [24] compared to the normal driving condition. The results of subjective fatigue evaluation in the present study indicate that the motion seat system was effective in reducing overall fatigue, mental fatigue, passive TR fatigue, drowsiness, and degradation in concentration, but not physical fatigue, and did not compromise driving safety.

The motion seat system could reduce passive TR fatigue by increasing cognitive demand on the driver by changing the driving posture passively. The motion seat system coordinating the backrest recline, cushion tilt, and lumbar support inflation/deflation along time changes the driving posture of the driver passively. Then, it can be assumed that the passively changed driving posture would activate the proprioceptors in the muscles and joints [51]. Lastly, it can be assumed that the activated signals of the proprioceptors stimulate the parietal lobe which recognizes the position and posture change of the whole body [52,53], which may reduce the level of monotony of the driver. The passive TR fatigue reduction mechanism of the motion seat system proposed in the present study can be also applicable to the effects of sensory stimulation methods such as the vibrating seat [49], haptic steering wheel [54], and facial cooling system [28] on mental fatigue.

The motion seat system in the present study which does not provide secondary tasks to the driver can be preferred to interactive technologies because it is less distracting during driving. Interactive technologies such as a speech-controlled game box system [26], common knowledge questions [9,10,27], and conversation [12,55], which assign a secondary task to the driver during driving have demonstrated their effectiveness in maintaining the driver’s alertness. However, Ried [56] reported that the driver might miss events hazardous to driving safety if the secondary task requires excessive attention from the driver. Therefore, Oron-Gilad et al. [9] stated that the development of secondary tasks that increase the overall demand on the driver without distracting the driver from the primary driving task is needed. The motion seat system would be preferred to the interactive technologies because the former has a lower possibility of distraction during driving than the latter requiring the active involvement of the driver.

The findings of the passive TR fatigue reduction of the motion seat system can be used to help the driver reduce the degradation of alertness in a partially autonomous vehicle or a long-haul transportation vehicle. Vehicle automation taking over the role of the steering wheel control and/or acceleration and deceleration control to the vehicle is effective to decrease active TR fatigue by reducing the task demands on the driver [24,57]. However, compared to manual control, partial automation (SAE level 2, [58]) or conditional automation (SAE level 3, [58]) requiring sustained monitoring by the driver can increase cognitive fatigue, brake response time, and steering wheel reaction time due to passive TR fatigue from increased monotony [14,24,57,59,60,61,62]. Next, previous studies have reported that occupational drivers of commercial vehicles such as heavy trucks and buses are more likely to be exposed to higher driver fatigue than non-commercial vehicle drivers due to prolonged driving periods and boredom in work conditions [63,64,65]. Therefore, the motion seat system can be applied as an effective countermeasure to mitigate passive TR fatigue while driving a partially autonomous vehicle and long-haul transportation vehicle.

The effect of the motion seat system on passive TR fatigue reduction needs to be further investigated to identify its generaliability for a large population and the effects of individual factors such as age, driving experience, and personality. The motion seat effectiveness for passive TR fatigue reduction identified with a small sample of 20 participants in the present study needs to be validated with a significantly large population representing drivers in various age, driving experience, and personality. Filtness et al. [66] and Otmani et al. [67] found that older drivers demonstrate better stability and less vulnerability while driving compared to young drivers in prolonged monotonous driving environments. Oron-Gilad et al. [9] proposed a driver’s workload model which distinguishes between active TR fatigue and passive TR fatigue considering the driver state as well as the situational demand. For example, low situational demand conditions such as driving on a monotonous highway or in a partially autonomous vehicle may cause passive TR fatigue to an experienced driver due to the underload condition but active TR fatigue to a novice driver due to the overload condition. Lastly, Thiffault and Bergeron [13] found that extroverted drivers were more sensitive to monotony than introverted drivers by showing a higher probability of fatigue-related driving errors in driving environments demanding low attention.

## Figures and Tables

**Table 1:** Criteria for driver fatigue status evaluation (adapted from Li et al. [29]).

Fatigue Status	Level	Features			
		Head	Eye	Gestures	Attention
Awake	1	- Keeping upright	- The eyes open widely - Blinking the eyes quickly - Moving the eyeballs actively - Closing the eyes briefly	- Single yawning - Single stretching	- Attentive to the outside
Drowsy	2	- Tilting/Shaking	- the eyelids half-closed - Moving the eyeballs slowly - Droopy eyelids - Blinking the eyes often	- Multiple yawning - Scratching the face - Swallowing - Sighing - Deep breathing - Rubbing the eyes - Multiple stretching - Moving around in the seat	- Decreased attention to the outside
Very drowsy	3	- Nodding	- Having trouble with keeping the eyes open - Blinking the eyes frequently - Closing the eyes for more than two sec. - Heavy eyelids	- Dozing/napping occasionally	- Almost losing the driving capability

**Table 2:** Driver’s fatigue assessment questionnaire.

Item			Description	Visual Analog Scale (VAS)
Overall fatigue			The degree of overall fatigue	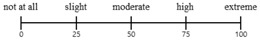
Physical fatigue			The degree of physical fatigue	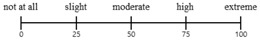
Mental fatigue			The degree of mental fatigue	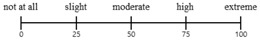
Mental fatigue	Active task-related fatigue		The degree of mental fatigue due to driving (e.g., lane change, brake pedaling)	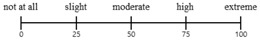
	Passive task-related fatigue		The degree of mental fatigue due to sustained attention	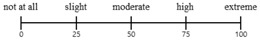
	Passive TR fatigue	Degradation indriving safety	The degree of fatigue interfering with driving safety	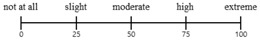
		Drowsiness	The degree of fatigue leading to drowsiness	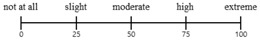
		Degradation in concentration	The degree of fatigue degrading concentration